# A Case Report of Folliculotropic Mycosis Fungoides: Diagnostic Criteria and Treatment Strategy

**DOI:** 10.7759/cureus.71994

**Published:** 2024-10-21

**Authors:** Tina Kituashvili, Tamari Urushadze, Sopiko Liluashvili, Ketevani Kankava, Nona Bagdadishvili

**Affiliations:** 1 Department of Dermatology, Ivane Javakhishvili Tbilisi State University, Tbilisi, GEO; 2 Department of Dermatology, Scientific/Research National Center of Dermatology and Venereology "Kanveni", Tbilisi, GEO; 3 Department of Dermatology, Kutaisi University, Tbilisi, GEO; 4 Department of Molecular and Medical Genetics, Tbilisi State Medical University, Tbilisi, GEO

**Keywords:** cicatricial alopecia, follicular mucinosis, folliculotropic mycosis fungoides, phototherapy, t-cell lymphoma

## Abstract

Folliculotropic mycosis fungoides (F-MF) is considered a unique variant of mycosis fungoides (MF), which is a form of cutaneous T-cell lymphomas (CTCLs). F-MF can appear in various forms, including patches, papules, plaques, nodules, and tumors. It often affects the face and extremities, with some cases involving the eyebrows. The distinction between F-MF and classical MF can be based on histopathology, which can demonstrate the involvement of hair follicles in the disease process. However, this differentiation can be complex. Immunohistochemical studies can sometimes assist in this distinction by showing differences in cell ratios. We describe a patient with hairless lesions and fine scaling, primarily located on the face, including eyebrow involvement, as well as on the extremities. Through a combination of clinical observations, dermoscopy, histopathology, and immunohistochemical analysis, a potential diagnosis of F-MF was considered. These diagnostic methods allowed us to accurately determine the disease subtype, aiding in both prognostic and therapeutic decisions.

## Introduction

Folliculotropic mycosis fungoides (F-MF) is a distinct variant and the most common subtype of mycosis fungoides (MF) characterized by specific clinicopathologic features. The folliculotropic pattern in F-MF, describing selective dermal infiltration of hair follicles by atypical lymphocytes, was first recognized by Piper in 1960 [1]. The term "follicular mycosis fungoides" was first used by Kim in 1985, based on the involvement of hair follicles in the disease process rather than the epidermis [2]. While F-MF was previously considered to have a more aggressive disease course, more recent studies report overall better survival and suggest a distinction between two prognostically different subtypes of F-MF, each with recognizable clinicopathological characteristics [3,4]. Based on the excellent prognosis of early-stage F-MF with a five-year overall survival of 92%, it is considered an indolent subtype of disease. In contrast, advanced-stage disease shows a 50% overall survival rate and is categorized as an aggressive variant [4]. The subclassification of F-MF contributed to the identification of new possible clinical, histological, and immunohistochemical prognostic factors for the disease that once again highlight the importance of clinicopathologic correlation regarding diagnosis [5,6]. In our paper, we describe a patient with F-FM, a case in which we used the aforementioned diagnostic differentiations between F-MF subtypes for the formation of the treatment plan.

## Case presentation

**Figure 1:**
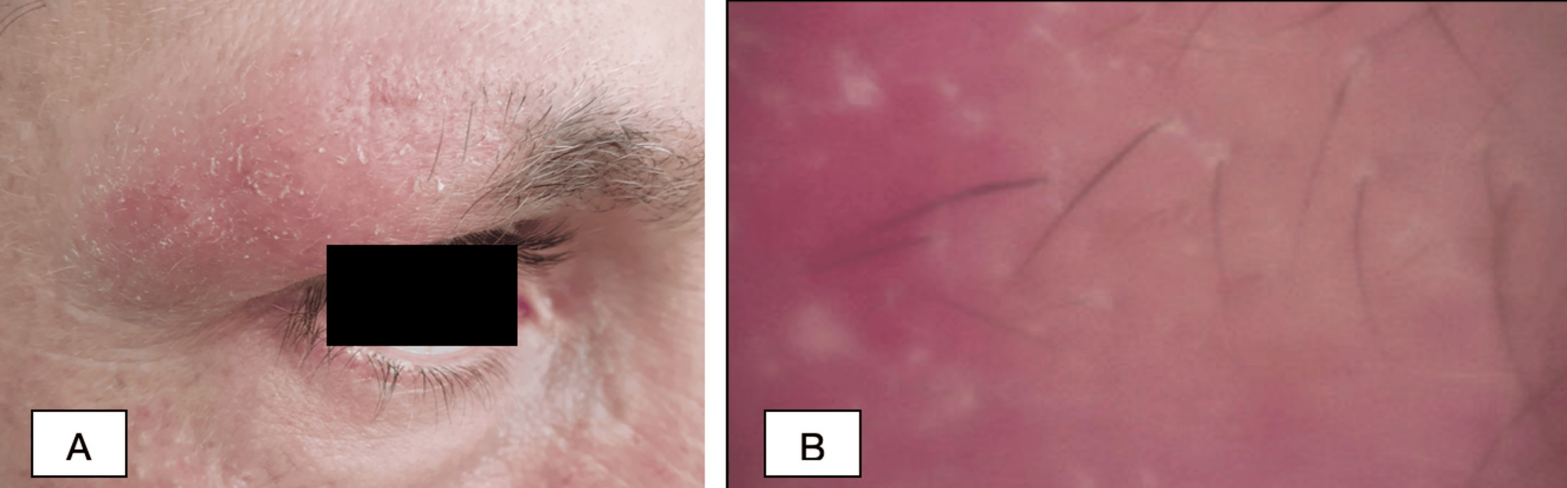
Representative pictures of the patient (A) Clinically, the half of the right eyebrow is faded and swollen, with erythema and fine scaling. (B) Dermoscopy shows perifollicular scaling with an erythematous background and a white structureless area.

**Figure 2:**
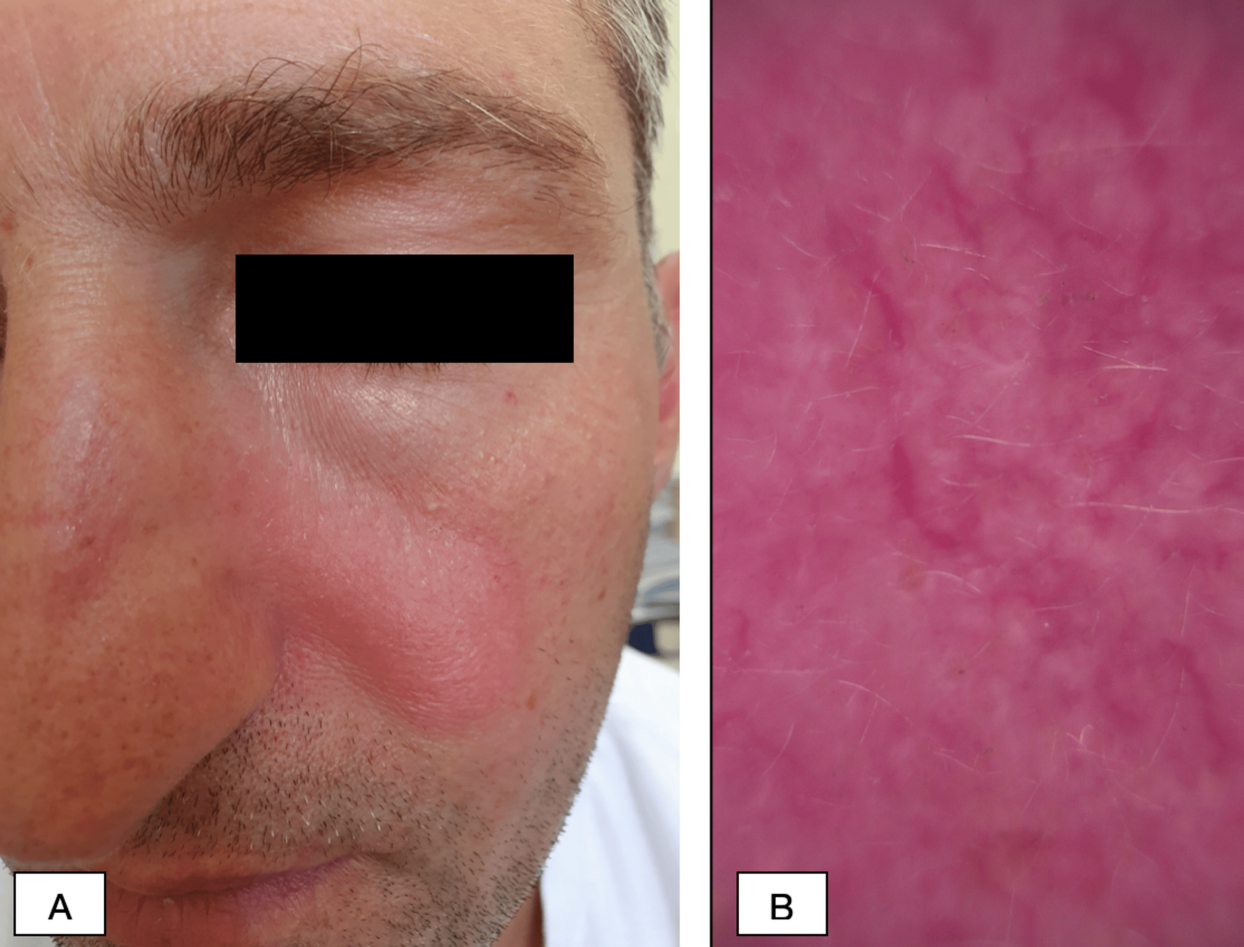
Representative pictures of the patient (A) Clinically, an erythematous macule 4-5 cm in size with little edema is seen on the left cheek. (B) Dermoscopy shows linear vessels on the left cheek.

**Figure 3:**
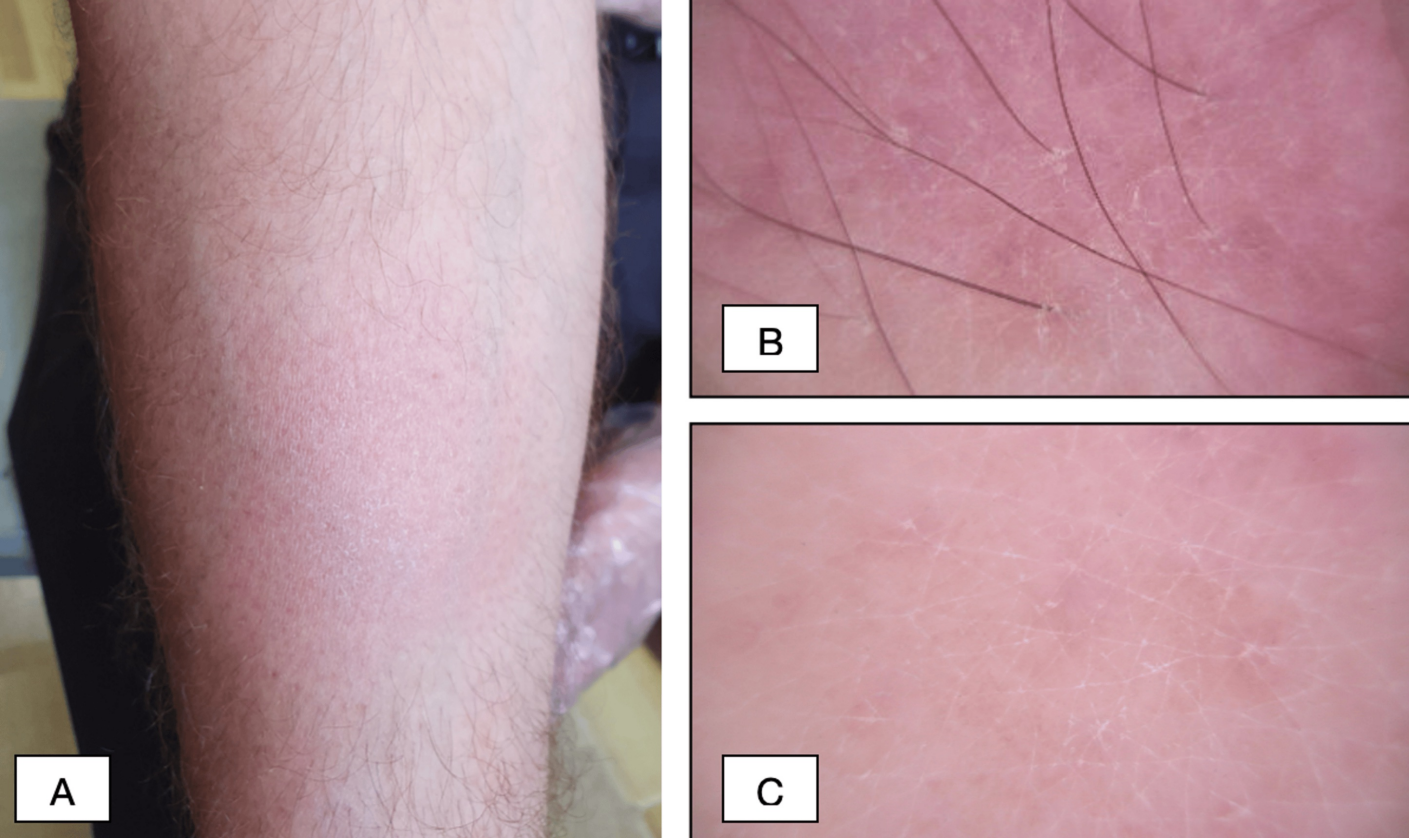
Representative pictures of the patient (A) Clinically, the hairless area with fine scaling is seen on the right forearm. (B, C) Dermoscopy of the lesion on the right forearm shows a fine perifollicular scaling.

**Figure 4:**
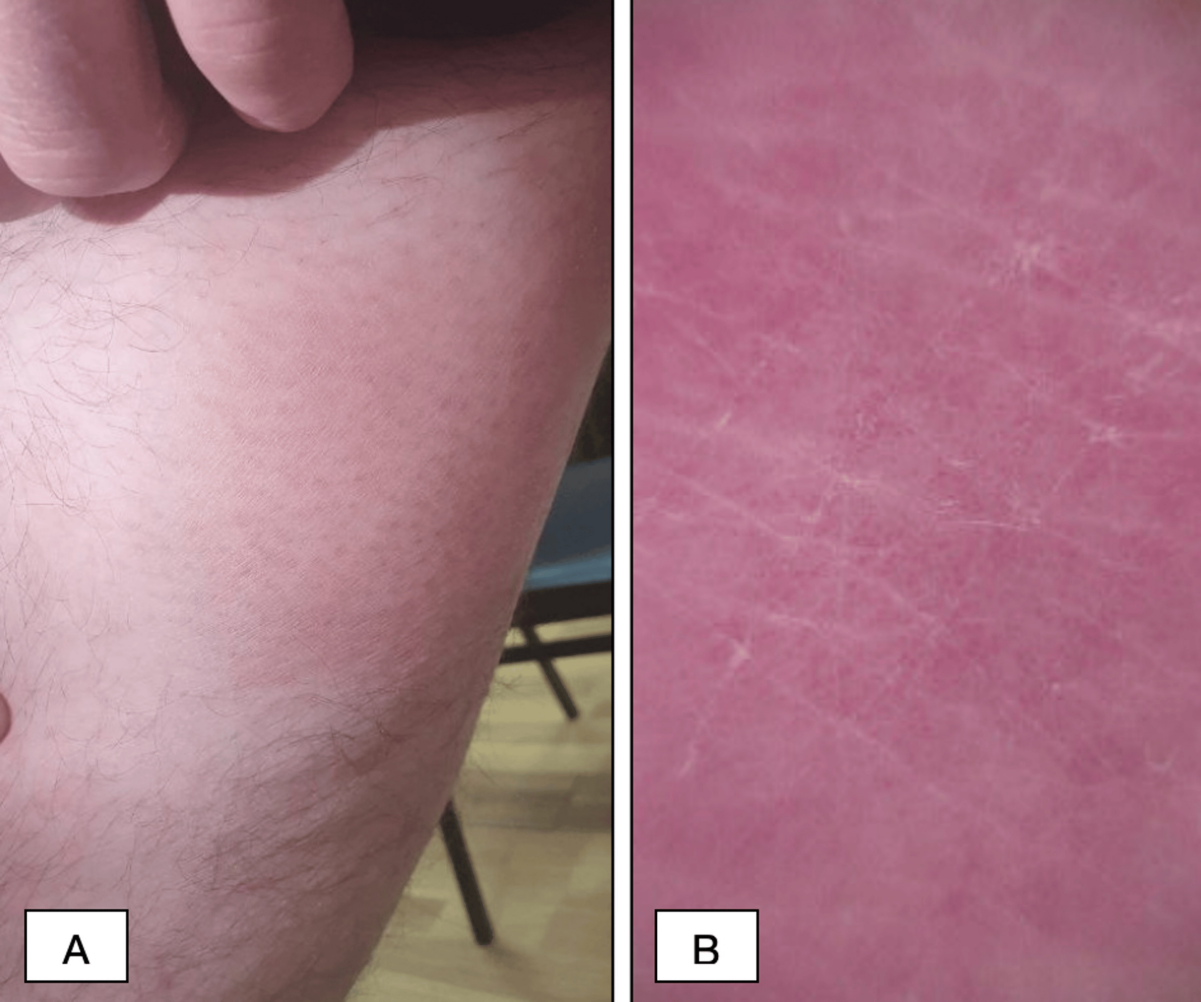
Representative pictures of the patient (A) Clinically, the hairless area with fine scaling is seen on the right thigh. (B) Dermoscopy visualizes dotted vessels and scaling.

A 39-year-old Caucasian male patient presented at the dermatology clinic with about a two-three-year history of rash on the face and extremities. The patient occasionally used local skin care products, without any improvement. On clinical examination, the half of the right eyebrow was faded and swollen, with erythema and fine scaling (Figure 1A). On the left cheek, there was an erythematous macule 4-5 cm in size with little edema (Figure 2A). The hairless areas with fine scaling were detected on both forearms, right shin, and right thigh (Figure 3A and Figure 4A). Manual palpation of the regional lymph nodes did not demonstrate lymphadenopathy.

In our clinic, a dermoscopic examination was made, which showed linear vessels on the left cheek (Figure 2B), perifollicular scaling with erythematous background and white structureless area on the right eyebrow (Figure 1B), fine perifollicular scaling on the right forearm (Figure 3B, 3C) and right shin, and dotted vessels on the right thigh (Figure 4B).

T-cell lymphoma was suspected clinically. To confirm the diagnosis, a biopsy was performed. Investigations were carried out and included a complete blood count (all parameters were normal), anti-nuclear antibodies, and lupus erythematosus (LE) cells, which were negative. Until the biopsy results were obtained, the patient was prescribed topical pimecrolimus 1% cream two times a day (BID) on every affected area.

**Figure 5:**
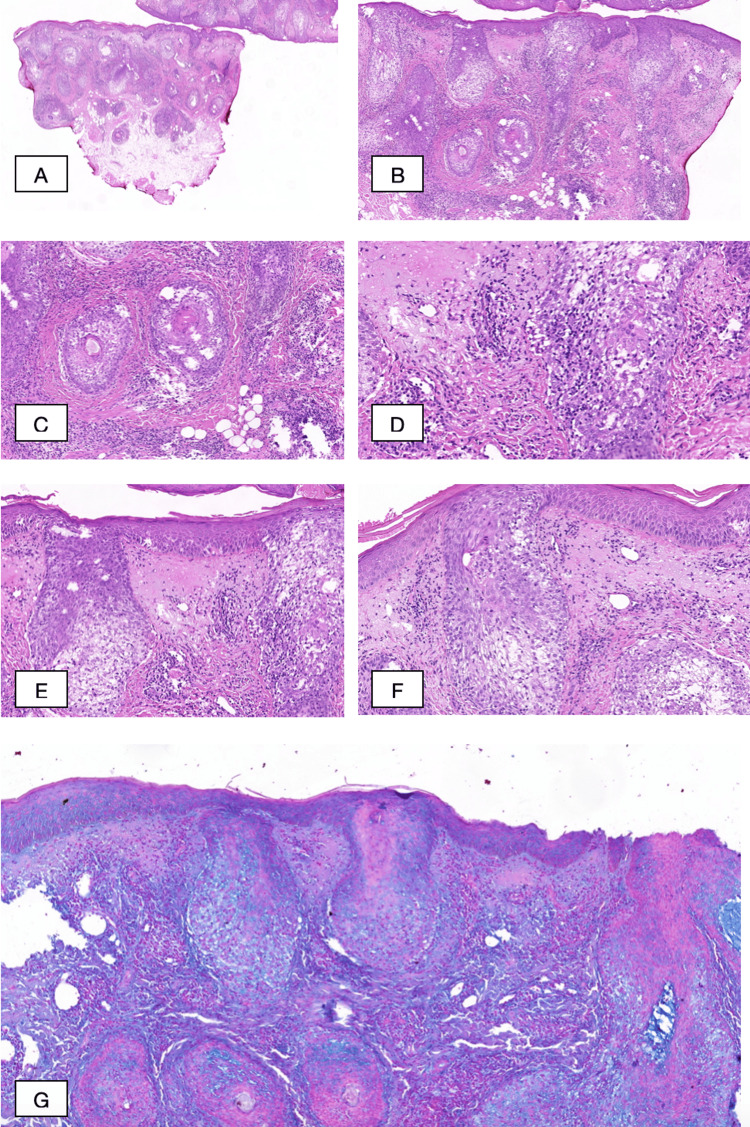
Histopathological examination of a cutaneous biopsy Histopathological examination shows excessive perifollicular lymphocytic infiltration with large lymphocyte nuclei and uneven contours, part of them with a "cerebriform" appearance. Pronounced follicular mucinosis is seen with mucinous infiltrates within the hair follicles throughout the dermis. The dermis is swollen. (A-F) Hematoxylin-eosin stain: (A) original magnification: ×2.2; (B) original magnification: ×5.6; (C) original magnification: ×10; (D) original magnification: ×20; (E) original magnification: ×13.1; and (F) original magnification: ×15. (G) Alcian blue stain: original magnification: ×7.8

The biopsy was taken from the right eyebrow (size: 0.5×0.5×0.3 cm) and from the right forearm (size: 0.7×0.5×0.5 cm). Histopathological examination of a skin biopsy specimen from the eyebrow showed excessive lymphocytic infiltration around the hair follicle and one area, where some of the lymphocytes were present within the follicular epithelium. Lymphocyte nuclei were large, and contours were uneven, part of them with a "cerebriform" appearance. Highly expressed follicular mucinosis with mucinous infiltrates was detected throughout the dermis. The dermis was swollen (Figure 5).

Compared to the above-described biopsy, a less pronounced perifollicular inflammation was observed in the biopsy specimen from the forearm. The accumulation of mucin in the follicles and dermis was visible in this area as well. Immunohistochemical analysis was performed, which revealed lymphocytic infiltration with the following phenotypes: CD3(+), CD5(+), CD43(+) and CD20(-), and CD38(-) and CD68(-). CD4-positive cells exceeded the level of CD8-positive lymphocytes. Ki-67 was positive in 5% of the cells.

**Figure 6:**
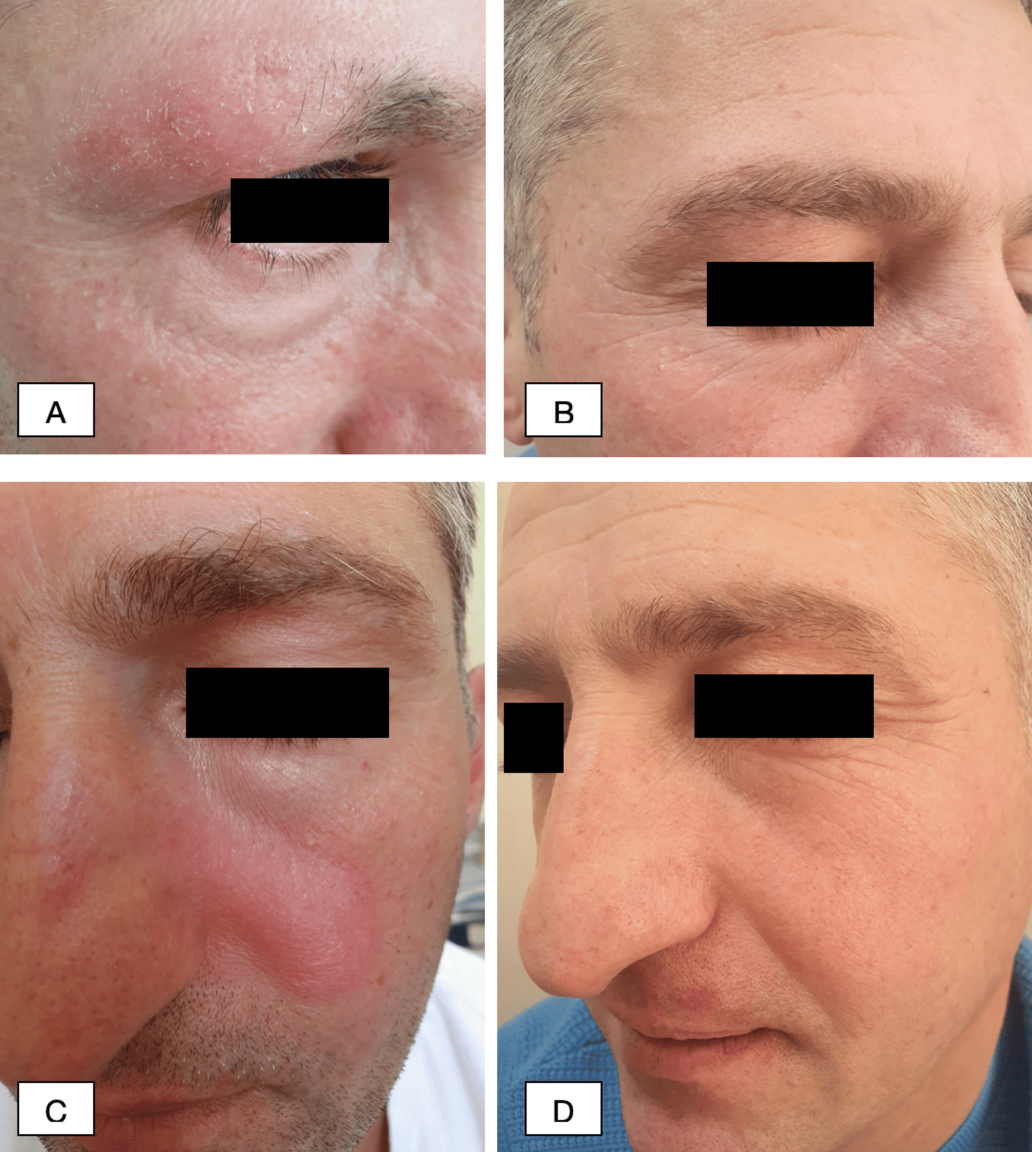
Representative pictures of the patient (A and C) First visit. (B and D) After 15 sessions of NB-UVB therapy. NB-UVB: narrowband ultraviolet B

On the basis of the clinical, dermoscopic, pathological, and immunohistochemical findings, the final diagnosis of F-MF with mucinosis was made. The patient was prescribed topical urea 5% daily, in the morning, and mometasone furoate 0.1% daily, in the evening. Narrowband ultraviolet B (NB-UVB) therapy was started with Waldmann UV 100L. The starting dose was 0.045 J/cm^2^ up to a maximal dose of 0.13 J/cm^2^. The patient did not undergo the whole course of treatment and stopped after 15 sessions of whole-body treatment. Despite this, significant improvement was observed: the edema and erythema on the face fully subsided, the hair on the right eyebrow grew back fully, and on other affected sites, the growth of vellus hairs was seen (Figure 6).

## Discussion

Primary cutaneous lymphomas are a heterogeneous group of cutaneous T-cell lymphomas (CTCLs) and cutaneous B-cell lymphomas (CBCLs) that present in the skin with no evidence of extracutaneous disease at the time of diagnosis [7]. MF and Sézary syndrome (SS) are the classic types of CTCLs, in which more than 60% of cases are attributed to MF [8]. According to the 2018 update of the World Health Organization (WHO)-European Organisation for Research and Treatment of Cancer (EORTC) classification for primary cutaneous lymphomas, aside from the classical form of Alibert-Bazin MF, there are three other distinct variants of MF: F-MF, pagetoid reticulosis, and granulomatous slack skin syndrome [7]. While classical MF accounts for about 40% of MF cases, F-MF presents in up to 5% of all MF cases and is more common at the age of 55-65 with male predominance [7,9].

F-MF is characterized by specific clinicopathologic features. It mainly presents with follicular papules, acneiform and milia-like lesions, erythematous plaques, nodules, and tumors, often with severe pruritus, preferentially affecting the face, head, and neck, with the highly characteristic clinical feature of eyebrow involvement [10,11]. In 81% of cases, follicular prominence is accompanied by alopecia, with or without scarring [3,4].

Clinically, F-MF can be differentiated into two stages: early and advanced. The early stage is characterized by follicle-based patches and acneiform or keratosis pilaris-like lesions. In the advanced-stage nodules, tumors, erythroderma, and/or extracutaneous manifestations can be seen [6]. Previously, F-MF was thought to be an aggressive form of MF [3]. However, more recent studies suggest a differentiation between indolent and aggressive F-MF based on the aforementioned two clinical stages [3,4]. There is still uncertainty regarding the categorization of plaques, which are considered to be one of the most frequent clinical features of F-MF [12]. Histopathological criteria should be used to distinguish between early plaque-stage F-MF and advanced plaque-stage F-MF [4]. The distribution also differs between stages. While advanced-stage lesions are mainly found on the face/neck, this location is less commonly affected in early-stage disease, which typically develops on the trunk/extremities [6,11]. Our patient had skin-limited lesions, interestingly including both thin erythematous plaques on the face and patches on the extremities.

The diagnosis of F-MF is based on histopathology, which reveals a dense inflammatory perifollicular infiltrate in the dermis, composed of medium-sized to large atypical T cells with cerebriform nuclei. Typically, the interfollicular epidermis is spared, with folliculotropism occurring instead of epidermotropism, the latter being associated with disease progression and reduced survival [10,13]. Pautrier's microabscesses are rarely seen [14,12]. In a few cases, histopathology demonstrates lymphocytic infiltrates around the eccrine glands, known as syringotropism [11,15].

A distinction can be made between plaques that are histologically characterized by sparse perifollicular infiltrates containing few, mainly small, neoplastic T cells (early plaque-stage F-MF) and plaques with more extensive, confluent, or diffuse infiltrates containing numerous medium- to large-sized tumor cells (advanced plaque-stage F-MF) [4]. In approximately 60% of cases, mucinous deposits in the follicular epithelium are detected, suggesting a classification of F-MF with or without associated follicular mucinosis [10]. The degree of follicular mucinosis does not differ between early- and advanced-stage F-MF, but the presence of prominent follicular mucinosis is thought to correlate with reduced disease progression and increased survival [6,13]. Our patient's biopsy specimen, taken from the plaque on the right eyebrow, demonstrated a dense perifollicular infiltrate with large-sized atypical lymphocytes and involvement of the epidermis in a single area. These findings more closely resembled advanced-stage F-MF. However, the higher degree of folliculotropism compared to epidermotropism and the detection of highly expressed follicular mucinosis contributed to the grading of the disease as an indolent form of F-MF.

Characteristic immunohistochemical findings of F-MF include an elevated intraepidermal CD4:CD8 ratio of 6 to 10:1, which can be linked to an extensive number of CD4+ Langerhans cells in the follicular epithelium [11,16]. A frequent loss of T-cell surface antigens, typical for classic MF (e.g., CD2, CD5, and/or CD7), has also been described [11]. An increased number of Ki-67-positive cells can be detected, with a higher count in the advanced stages of F-MF. Some studies have demonstrated that the presence of more than 10% Ki-67-positive cells could be used as a negative prognostic factor, as they are associated with increased disease progression [13]. Additionally, an increased number of CD20+ B cells and CD68+ macrophages have been observed, with significantly higher expression in advanced-stage F-MF [5,13]. Our assessment of the grading of our patient's disease was reinforced by the immunohistochemical findings: detected low Ki-67 expression in the absence of CD20 and CD68 surface antigens could be considered positive prognostic factors, helping to shape our upcoming therapeutic strategy.

While many specific clinicopathologic patterns of F-MF have been described, pathognomonic dermoscopic features and, therefore, the prognostic value of dermoscopy in F-MF are yet to be fully identified. However, a few small studies have outlined overlapping dermoscopic features, such as perifollicular accentuation seen as a white halo around the follicle, perifollicular scaling, comedo-like openings, white structureless areas replacing lost hair follicles, dotted, fine short linear and glomerular vessels, black dots, or broken hairs [17,18]. In the case of our patient, dermoscopy revealed linear vessels on the left cheek, perifollicular scaling with an erythematous background and a white structureless area on the right eyebrow, fine perifollicular scaling on the right forearm and right shin, and dotted vessels on the right thigh. In our case, dermoscopy was used as a relatively inexpensive and, more importantly, fast tool to evaluate the lesion.

There are no globally accepted treatment guidelines for F-MF, and the existing data are primarily based on studies and case reports. Treatment options for F-MF are similar to those for classic MF and include skin-directed therapies (SDTs), such as topical drugs, phototherapy, radiotherapy, and various systemic agents [11]. However, F-MF tends to be more resistant to commonly used therapeutic options [10]. It has been proposed that the poor response to SDTs may be due to the depth and location of the lymphocyte infiltrates [11]. The mechanisms of action for systemic treatments have not yet been fully understood.

The optimal treatment for F-MF is still undefined. In the meantime, phototherapy-based treatment (psoralen+ultraviolet A (PUVA) or UVB), radiation therapy (local or total skin electron beam therapy (TSEB)), topical corticosteroids, and oral retinoids are the most commonly prescribed therapies, with varying results [19]. According to a 2017 study by the Dutch Cutaneous Lymphoma Group involving 203 patients from 1985 to 2014, early- and advanced-stage F-MF showed different treatment responses, supporting the idea of tailored therapeutic strategies for each variant [20]. While early-stage F-MF responded to monotherapy with nonaggressive SDTs, the same outcomes were not observed in patients with advanced-stage disease [20]. Additionally, a large real-life study revealed that disease characteristics, such as the presence of patch/flat plaque or thick plaques in MF or F-MF, influence treatment strategies [21]. Earlier studies suggested that PUVA phototherapy was more effective than NB-UVB. However, recent studies have suggested a distinction in indications for these treatments, with NB-UVB being more suitable for patch/thin plaque F-MF and PUVA for thick plaque disease [20,21].

After the diagnosis and disease stage were confirmed, we considered recent findings and initiated treatment with NB-UVB phototherapy, along with topical mometasone furoate and urea. After 15 sessions of phototherapy, our patient showed significant improvement. Despite the lack of long-term follow-up, this case demonstrates that the described diagnostic criteria can have prognostic value in F-MF and can be highly useful in determining the course of treatment.

## Conclusions

Our paper presents a case where the staging of F-MF was evaluated according to existing diagnostic criteria, including clinical, dermoscopic, histological, and immunohistochemical features. The presence of thin erythematous plaques on the face and patches on the extremities, more pronounced folliculotropism compared to epidermotropism, and highly expressed follicular mucinosis on histopathology, along with low Ki-67 expression and the absence of CD20 and CD68 on immunohistochemistry, contributed to classifying the disease as an indolent form of F-MF. Disease staging, combined with existing data on the therapeutic approaches guided by diagnostic criteria, was used to choose an optimal management plan, which proved to be successful.
